# First-in-Human Prospective, Observational, and Comparative Clinical Study of Simultaneous Invasive and Non-Invasive Intracranial Pressure Pulse Wave Monitoring

**DOI:** 10.3390/s26051403

**Published:** 2026-02-24

**Authors:** Indre Lapinskiene, Edvinas Chaleckas, Vilma Putnynaite, Laimonas Bartusis, Yasin Hamarat, Aidanas Preiksaitis, Mindaugas Serpytis, Vytautas Petkus, Saulius Vosylius, Arminas Ragauskas

**Affiliations:** 1 Clinic of Anaesthesiology and Intensive Care, Institute of Clinical Medicine, Faculty of Medicine, Vilnius University, LT-03101 Vilnius, Lithuania; indre.lapinskiene@santa.lt (I.L.); mindaugas.serpytis@santa.lt (M.S.); saulius.vosylius@rvul.lt (S.V.); 2 Vilnius University Hospital Santaros Klinikos, LT-08406 Vilnius, Lithuania; 3 Health Telematics Science Institute, Kaunas University of Technology, LT-51423 Kaunas, Lithuania; edvinas.chaleckas@ktu.lt (E.C.); vilma.putnynaite@ktu.lt (V.P.); laimonas.bartusis@ktu.lt (L.B.); vytautas.petkus@ktu.lt (V.P.); arminas.ragauskas@ktu.lt (A.R.); 4 Laboratory of Heat-Equipment Research and Testing, Lithuanian Energy Institute, LT-44403 Kaunas, Lithuania; 5 Clinic of Neurology and Neurosurgery, Institute of Clinical Medicine, Faculty of Medicine, Vilnius University, LT-03101 Vilnius, Lithuania; aidanas.preiksaitis@santa.lt

**Keywords:** intracranial pressure, non-invasive monitoring, intracranial compliance, traumatic brain injury, subarachnoid hemorrhage, intracerebral hemorrhage

## Abstract

**Highlights:**

**What are the main findings?**

- The novel, fully passive, non-invasive intracranial pressure pulse wave monitoring system (Archimedes 02) demonstrated a strong association with invasive ICP measurements and was able to capture key features of ICP pulse wave morphology.
- The non-invasive system detected P2/P1 ratios, which reflect intracranial compliance, in a manner consistent with invasive ICP monitoring, indicating that it can capture relevant intracranial dynamics.

**What are the implications of the main findings?**

- The Archimedes 02 technology represents a promising, safe, real-time non-invasive approach to ICP pulse wave assessment, potentially reducing complications associated with invasive monitoring such as infection, hemorrhage, and sensor drift.
- By enabling continuous non-invasive assessment of intracranial compliance, this approach may improve neurocritical care management and expand access to ICP monitoring in settings where invasive techniques are impractical.

**Abstract:**

Monitoring intracranial pressure (ICP) dynamics is critical for the management of traumatic brain injury, stroke, other neurosurgical conditions, and cerebral blood flow autoregulation; however, invasive ICP monitoring carries risks such as infection, hemorrhage, and sensor zero drift. Increasing evidence suggests that ICP waveform morphology provides clinically relevant information beyond mean ICP value alone. In this first-in-human prospective comparative clinical study, we evaluated the feasibility and accuracy of a novel, fully passive, non-invasive ICP pulse waveform monitoring system (Archimedes 02) based on the detection of eyeball mechanical movement. Fifteen intensive care unit patients (6 males, 9 females; mean age 57.1 ± 18.8 years) with clinically indicated invasive ICP monitoring or external ventricular drainage were enrolled. Three-minute monitoring sessions were performed to simultaneously acquire non-invasive ICP pulse waveforms, invasive ICP waveforms, and invasive radial artery blood pressure (ABP) waveforms. Averaged waveforms were derived for each patient and compared graphically and using correlation analysis. Non-invasive ICP pulse waves recorded with Archimedes 02 showed a strong correlation with invasive ICP waveforms ($\bar{R}$ = 0.965). In contrast, correlations between non-invasive ICP and ABP waveforms ($\bar{R}$ = 0.699), as well as between invasive ICP and ABP waveforms ($\bar{R}$ = 0.749), were lower. These findings indicate that the non-invasive signal primarily reflects ICP dynamics rather than arterial blood pressure. This novel non-invasive ICP monitoring approach has the potential to enhance neurocritical care, particularly in settings where invasive monitoring is impractical or unavailable. Further validation in larger and more diverse patient populations is warranted.

## 1. Introduction

Intracranial pressure (ICP) is commonly monitored in intensive care units (ICUs) in patients with traumatic brain injury (TBI) or stroke, or following surgical tumor removal. Current guidelines recommend maintaining ICP below 22 mmHg, as higher values are associated with increased mortality [1]; however, invasive ICP measurement, considered the clinical standard for treatment decisions, carries potential risks, including infection, postprocedural hemorrhage, and sensor misplacement [2]. In addition, invasive ICP sensors’ zero drift depends on monitoring duration and can range from 1 to 5 mmHg [3]. This technique is also limited in situations where a cerebral autoregulation assessment is crucial, such as cardiac surgery, organ transplantation, extracorporeal blood flow support in ICUs, and aerospace medicine.

Among current invasive modalities, parenchymal ICP sensors provide direct, continuous measurement of brain tissue pressure and therefore capture intracranial pulsatility with high temporal fidelity. In contrast, external ventricular drains (EVDs) measure pressure within the ventricular cerebrospinal fluid (CSF), which may vary depending on catheter placement, CSF hydrodynamics, and ventricular compliance differences [4,5]. These physiological and technical distinctions can lead to characteristic waveform discrepancies between modalities and are important considerations when evaluating the agreement and clinical validity of emerging non-invasive ICP technologies.

In 1975, Anthony Marmarou described intracranial compliance (ICC) as the ratio between changes in intracranial volume (ΔV) and intracranial pressure (ΔICP), denoted as ICC = ΔV/ΔICP [6]. Up to a patient-specific threshold, ICC is high, and an increase in intracranial volume produces only a small rise in ICP. Beyond this specific limit, as the available intracranial space becomes constrained, ICC decreases, and even small volume increases can lead to dangerous ICP elevations, which may cause cerebral ischemia, potentially resulting in severe neurological deficits or death [7]. Conversely, excessively low ICP and abnormally high compliance have been associated with normal-tension glaucoma [8]. ICC is influenced by factors including arterial smooth muscle tone, the partial pressure of CO_2_, endothelial function, brain hydration, and metabolism [9]. It can also vary with body position, and across the day and night cycle [10].

Growing evidence suggests that the ICP pulse waveform provides more diagnostic information to guide clinical decision-making than the mean ICP value alone [11,12,13,14]. Importantly, ICC may be reduced even when mean ICP remains within normal limits, highlighting the value of waveform analysis for detecting intracranial pathology at an early stage [15]. Studies have shown that ICC monitoring can improve patient outcomes [16,17]. Because ICC directly determines the relationship between intracranial volume changes and pressure pulsatility, several studies have shown that ICP waveform morphology—particularly the relative amplitude of its characteristic peaks—offers a clinically relevant surrogate for intracranial compliance [18,19]. The ICP pulse wave is phased with the cardiac cycle and when ICP and ICC are within normal ranges, it typically has three morphological peaks: P1, P2, and P3. Changes in mean ICP and ICC alter both the pulse wave’s shape and amplitude; specifically, with decreasing ICC, the pulse wave becomes more rounded due to a relative increase in the P2 peak [20]. Accordingly, the ratio between the second and first peaks (P2/P1) has been proposed as a quantitative index of intracranial compliance, with values approaching or exceeding 1 (P2 ≥ P1) indicating reduced compliance [21,22]. Importantly, because these waveform features arise from the brain’s mechanical response to pulsatile arterial inflow, clinically meaningful information about ICC can be extracted even without calibrated absolute ICP values, which supports the diagnostic value of waveform-only monitoring approaches. Also, the ICP waveform can change due to age-related cerebrovascular system stiffening [23]. Invasive ICP sensors placed in the brain parenchyma or cerebral ventricles have been shown to record different pulse wave morphologies, as illustrated in a past case study [4].

The CSF is distributed across the cerebral ventricles, subarachnoid space, perivascular spaces, brain, spine, and optic nerve [24]. Optical coherence tomography (OCT) studies have shown that the optic nerve head pulsates with an amplitude of 7.8 ± 1.3 µm [25], and magnetic resonance imaging (MRI) has demonstrated that the human eye pulsates across all physiological frequency bands, including slow wave, respiratory, and cardiac cycles [26]. The results of a pilot study further indicated that there is no correlation between intraocular pressure (IOP) and the amplitude of pulsatile optic nerve head displacement [27].

We hypothesize that this eyeball mechanical movement phenomenon, which directly follows ICP pulsations, can be exploited to develop a novel, non-invasive, wireless sensor capable of real-time ICP change monitoring, including pulse wave morphology. Such a system could provide surrogate information about intracranial compliance even when absolute ICP values are unavailable or cannot be calibrated. Accordingly, we aimed to design and develop this monitoring system to test it in a first-in-human prospective, observational, and comparative clinical study using patients with implanted invasive ICP sensors, and to assess its feasibility, accuracy, and potential clinical use in monitoring ICP pulse waves and intracranial compliance.

## 2. Materials and Methods

### 2.1. Archimedes 02—Non-Invasive Intracranial Pressure Pulse Wave Monitor

Archimedes 02 is a novel, non-invasive, fully passive device designed to monitor ICP pulse waves by detecting the mechanical pulsatile motion of the eyeball beneath a closed eyelid. The monitor has a goggle-like design and is gently and securely positioned on the subject’s closed eyelids during measurement. Front and back views of the device, together with a detailed technical description, were provided in our previous pilot study conducted on healthy volunteers [28].

**Figure 1:**
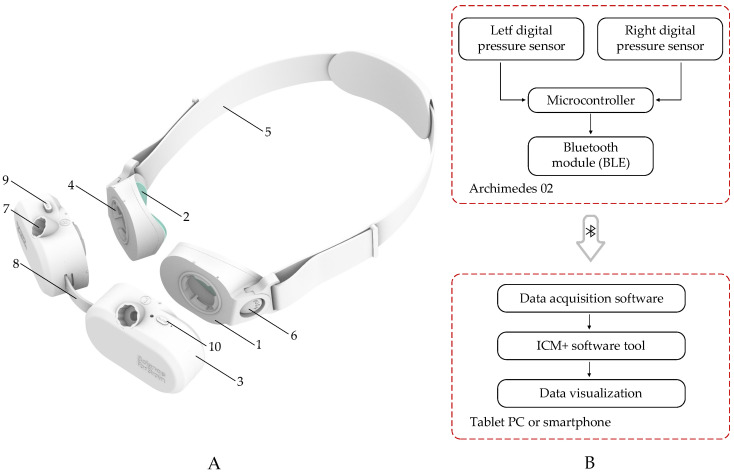
Archimedes 02 non-invasive intracranial pressure waveform monitoring system. (**A**)—Three-dimensional view of the device. 1—disposable cup; 2—elastic film; 3—sensor module; 4—locking mechanism for connecting the disposable cup to the sensor module; 5—headband; 6—buckle for connecting the headband to the disposable cups; 7—liquid injection port; 8—bridge; 9—bridge length adjustment button; 10—power button. (**B**)—Simplified block diagram of the monitoring system and signal acquisition diagram.

The device consists of two disposable cups designed for the left and right eyes, each separated from the eyelid by a thin, non-allergenic elastic film (approximately 20 µm thick in the most recent version), which acts as a barrier and prevents direct contact with the eyelid. Sensor modules can be easily connected to and disconnected from the top of these cups, while the device is secured on the head using an adjustable-length headband that attaches to the sides of the cups. The most recent version of the system is shown in Figure 1.

When the sensor modules are hermetically sealed onto the disposable cups, the internal volume of each cup is filled with an incompressible liquid through dedicated injection ports. The device operates on the principle that CSF pulsations within the optic nerve subarachnoid space induce corresponding micromechanical movements of the eyeball. These movements are detected by a high-resolution pressure sensor embedded within each sensor module, with the sensor port protruding into the cup and making direct contact with the liquid.

The digital pressure sensor is capable of measuring liquid pressure over a range of 0 to 51.715 mmHg with a resolution of 0.00316 mmHg. The Archimedes 02 intracranial pressure pulse wave monitor is powered by an internal rechargeable lithium-ion battery (LP402025JU, Jauch Quartz GmbH, Villingen-Schwenningen, Germany) and, once powered on, wirelessly transmits real-time data from the left and right sensors separately via a Bluetooth Low Energy (BLE) module (BLUENRG-M0L, STMicroelectronics, Plan-les-Ouates, Switzerland) to a laptop or smartphone.

The system is lightweight (228 g, including both sensor modules with liquid-filled cups) and passive, meaning that it does not emit any physical signals that could affect the eye, orbit, or intracranial space, nor does it apply additional pressure to the eye or surrounding structures. The goggle-like design, combined with disposable patient-contact components, makes the system cost-effective, while the dual-eye configuration enables the detection of potential asymmetries in intracranial pressure pulse waves between brain hemispheres. This feature may be useful for assessing conditions such as traumatic brain injury, stroke, intracranial vasospasm, and recurrent brain tumors.

### 2.2. Ethical Approvals

Ethical approval was obtained to conduct a first-in-human, prospective, comparative, observational clinical study (2024–2025) to provide clinical validation of the Archimedes 02 technology. Clinical data were collection in accordance with protocols approved by the Vilnius (Lithuania) Regional Biomedical Research Ethics Committee (no. 2024/3−1570−1030, 5 March 2024) in brain injury patients with implanted ICP sensors.

### 2.3. Data Collection and Analysis

For ICU patients, non-invasive ICP pulse wave data were recorded using the Archimedes 02 system, and invasive ICP data were measured using Raumedic Neurovent-PTO sensors, Codman sensors, or external ventricular drainage (EVD) systems. Arterial blood pressure (ABP) pulse wave data were also recorded when invasive radial artery monitoring was available. All data were collected using ICM+ software (version 9.1, Cambridge, UK) at a sampling frequency of 100 Hz, with each monitoring session lasting 3 min.

After data collection, signal processing and analysis were performed using MATLAB software (version R2024a, MathWorks, Natick, MA, USA). The raw pressure signals underwent a third-order Butterworth bandpass filter (0.5–8 Hz) to remove offsets, slow drifts, and respiratory components, isolating cardiac-related pulsations. Diastolic points were then identified using MATLAB’s *findpeaks* function, enabling precise segmentation of the continuous signal into individual pulse waves. Each segmented pulse was detrended by subtracting the linear baseline between the first and last sample, thereby removing low-frequency slope artifacts and standardizing the waveform to a zero-reference level. The pulses were then interpolated or decimated to a uniform length of 100 data points to facilitate consistent morphological comparison across beats and between invasive and non-invasive measurements. Waveforms with visible artifacts were excluded to ensure that only physiologically meaningful pulses contributed to the average waveform.

Following these steps, all valid pulses within each 3-min session were time-normalized and averaged to generate a representative ICP pulse waveform for that session. This processing pipeline mirrored the methodology used in our previous study [28], where the same approach was applied to derive average ICP pulse waves.

The averaged invasive ICP, non-invasive ICP (Archimedes 02), and ABP waveforms were compared by plotting all three pulse waveforms, normalized in time and amplitude, on the same axis for each patient. The P2/P1 ratio was calculated for both invasive and non-invasive ICP waveforms for each patient, and median values with interquartile ranges were computed across all patients. Pearson’s correlation coefficients (R) and the corresponding Fisher’s z-transformed values were calculated to evaluate waveform associations for each patient. Mean correlation coefficients ($\bar{R}$), obtained by back-transforming the averaged Fisher’s z values, were also estimated.

## 3. Results

**Figure 2:**
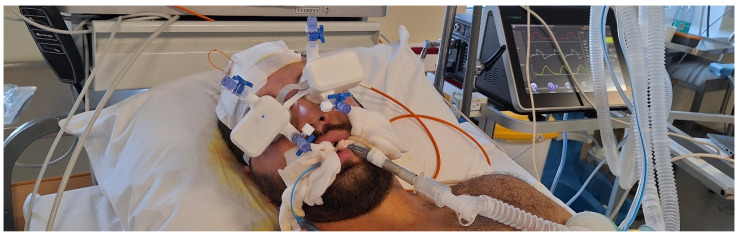
Simultaneous real-time invasive (Raumedic) and non-invasive (Archimedes 02) ICP pulse wave monitoring in the neurosurgical ICU.

Simultaneous invasive and non-invasive ICP monitoring, together with recording of the invasive ABP signal, was performed in patients undergoing treatment for subarachnoid hemorrhage, intracerebral hemorrhage, ruptured intracranial aneurysm, or intracranial meningioma. Data was recorded simultaneously for 3 min to derive an averaged pulse waveform for each monitoring session. Figure 2 illustrates the device in use during monitoring.

Fifteen ICU patients were included in this comparative study, each of whom required clinically indicated placement of an invasive ICP sensor or an external ventricular drain. The study cohort included one patient with a ruptured intracranial aneurysm, four with subarachnoid hemorrhage, five with traumatic brain injury, three following brain tumor removal, and two with intracerebral hemorrhage. Among the patients with TBI, one sustained injury in a road traffic accident, while the remaining four were injured due to falls. None of the fifteen patients included in this study required pleural drainage or experienced muscle paralysis.

The patients ranged in age from 20 to 85 years, with a mean age of 57.07 ± 18.81 years and included nine female and six male patients. Parenchymal ICP sensors were implanted in ten patients, and external ventricular drains were used in the remaining five. The average Glasgow Coma Scale (GCS) score was 8.33 ± 3.59, and the average Glasgow Outcome Scale (GOS) score was 2.73 ± 1.39. The characteristics of the individual patients are presented in Table 1.

**Figure 3:**
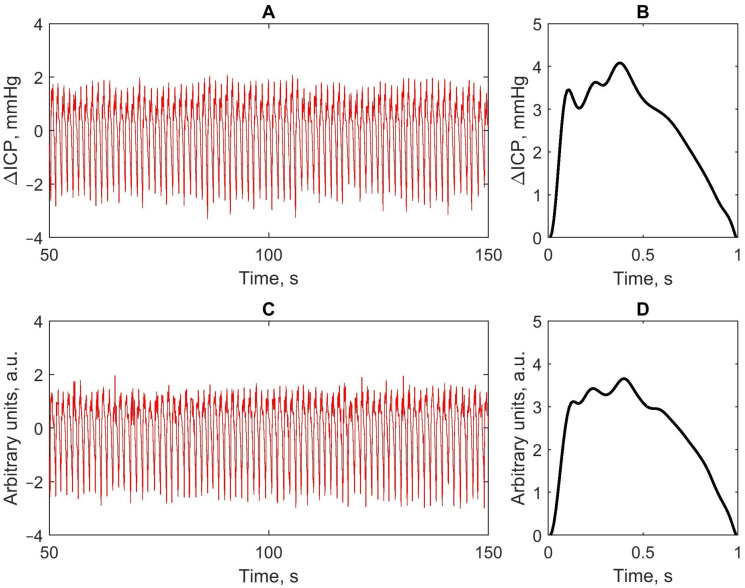
Example of a continuous invasive ICP pulse wave recording (**A**) with its 3-min averaged pulse wave (**B**), and a continuous non-invasive ICP pulse wave recording (**C**) with its 3-min averaged pulse wave (**D**).

A total of 15 monitoring sessions, each 3 min in duration, were analyzed, and each session was treated as an independent statistical unit. Figure 3 provides an example of invasive and non-invasive ICP pulse waves recorded from a single patient.

**Figure 4:**
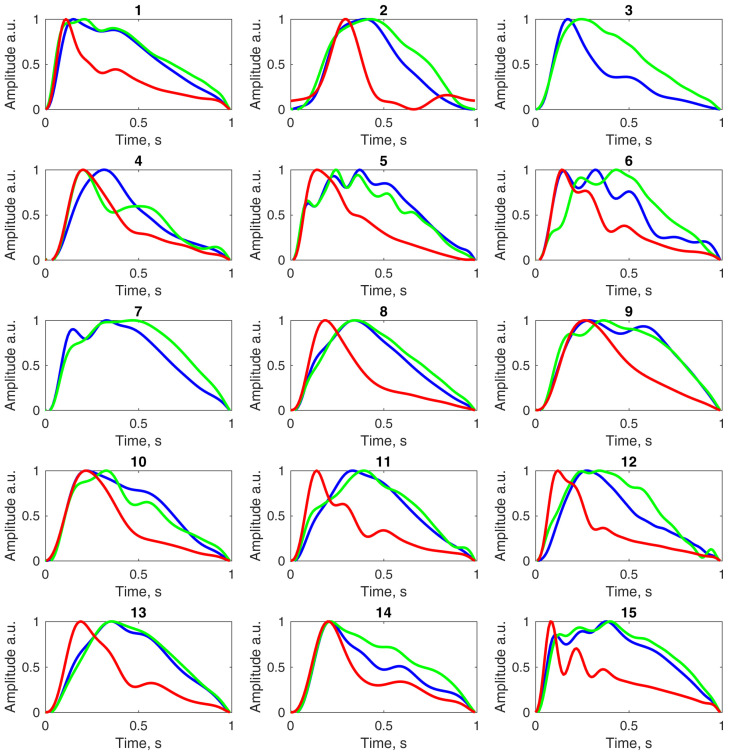
Averaged and normalized pulse waveforms of invasive ICP (blue), non-invasive ICP obtained with the Archimedes 02 device (green), and invasive ABP (red), recorded simultaneously. The number above each signal panel indicates the corresponding ICU patient.

The calculated Pearson’s correlation coefficient between the averaged invasive and non-invasive ICP pulse waves shown in Figure 3 was R = 0.982. Figure 4 presents the averaged invasive and non-invasive ICP pulse waveforms, along with the averaged ABP waveform, computed separately for each of the 15 ICU patients to facilitate visual comparison of waveform morphology. Arterial blood pressure pulse waves for the third and seventh patients are not shown because ABP recordings for these patients were unavailable due to technical issues.

Associations between invasive and non-invasive ICP pulse waveforms, between non-invasive ICP and invasive ABP waveforms, and between invasive ICP and ABP waveforms are presented as Pearson’s correlation coefficients and Fisher’s z-transformed values in Table 2. Data for non-invasive ICP versus ABP, and invasive ICP versus ABP are missing for the third and seventh patients because ABP recordings were unavailable. Calculated P2/P1 ratios for invasive and non-invasive ICP pulse waveform for each ICU patient are also provided in Table 2.

The median P2/P1 ratio of the averaged invasive ICP pulse waveform across all 15 ICU patients was 1.013 (IQR: 0.853–1.243), while for the non-invasive ICP waveform it was 1.099 (IQR: 0.878–1.480).

Among all 15 ICU patients, median Fisher’s z values and corresponding mean correlation coefficients were as follows: invasive versus non-invasive ICP, 2.227 (IQR: 1.753–2.351) and $\bar{R}$ = 0.965; non-invasive ICP versus invasive ABP, 0.636 (IQR: 0.551–1.154) and $\bar{R}$ = 0.699; and invasive ICP versus invasive ABP, 0.804 (IQR: 0.707–1.201) and $\bar{R}$ = 0.749.

## 4. Discussion

The human optic nerve is surrounded by cerebrospinal fluid within its subarachnoid space, providing a unique anatomical pathway that enables non-invasive access to ICP dynamics. The optic nerve head (ONH), pulsating with an amplitude of approximately 7.8 ± 1.3 μm, provides the physiological basis for leveraging ocular biomechanics in non-invasive ICP waveform assessments [25]. Advances in optical coherence tomography (OCT) and functional magnetic resonance imaging (fMRI) have demonstrated measurable human eye movements across multiple physiological bands, further supporting the feasibility of detecting ICP pulsations via eyeball spatial movement [26]. Importantly, a previous study found no correlation between intraocular pressure (IOP) and ONH pulsation amplitude, suggesting that these movements primarily reflect ICP [27]. The clinical relevance of ICP waveform morphology has motivated the search for novel non-invasive modalities capable of capturing these dynamics [11].

Building on this physiological and technological rationale, we conducted a first-in-human comparative study in ICU patients with invasive ICP sensors to evaluate a recently developed non-invasive ICP pulse waveform monitor, Archimedes 02, that passively detects eyeball mechanical movement.

Our results demonstrated a high correlation between non-invasive ICP pulse waves recorded with the Archimedes 02 and reference invasive ICP readings ($\bar{R}$ = 0.965). In contrast, correlations between non-invasive ICP waveforms and invasive arterial blood pressure waveforms ($\bar{R}$ = 0.699), and between invasive ICP and ABP waveforms ($\bar{R}$ = 0.749), were lower. These findings suggest that the non-invasively detected pulse waves primarily reflect ICP dynamics rather than ABP. Additionally, the median P2/P1 ratio derived from averaged non-invasive waveforms (P2/P1 = 1.099) was in close agreement with the median ratio calculated from invasive ICP data (P2/P1 = 1.013), supporting the potential of this non-invasive method to capture ICP pulse wave morphology with clinically relevant accuracy.

Monitoring sessions in this study lasted three minutes, an interval sufficient to capture at least one full cycle of slow ICP waves, which range from approximately 20 to 200 s [29,30]. Shorter, controlled recordings minimized variability due to changes in the patient’s physiological state, ensuring robust comparative analysis of ICP pulse wave morphology.

All patients included in this study were comatose and either mechanically ventilated or breathing spontaneously. Sedation and ventilation strategies varied according to clinical requirements, ranging from no sedation to deep sedation (RASS −5), and multiple mechanical ventilation modes were used (ASV, VC-SIMV, CPAP, VC-CMV, VC-AC). Additionally, the patient who sustained a traumatic brain injury from a road traffic accident, as well as all other patients, did not require pleural drainage. The absence of pleural drainage across the cohort likely minimized confounding influences on ICP waveform measurements. Future studies should consider standardizing sedation and ventilation protocols to further validate non-invasive ICP pulse wave monitoring technology under controlled conditions.

The development of a non-invasive, real-time ICP pulse waveform monitor based on the eyeball’s mechanical movements holds considerable promise. Such a device could eliminate the risks associated with invasive monitoring, including infection and hemorrhage [2], while providing continuous data on ICP dynamics. This approach would be particularly advantageous in settings where invasive monitoring is contraindicated, impractical, or unavailable, such as in low-resource environments or for long-term monitoring of chronic conditions.

Finally, although the Archimedes 02 demonstrated high correlation with invasive ICP readings in this first-in-human comparative clinical study, its performance in patients with abnormal intracranial anatomy or severe brain injury remains to be fully evaluated. Factors such as age, intracranial compliance, and underlying pathology may influence the accuracy of existing non-invasive ICP monitoring techniques [9]. The fully passive design of the Archimedes 02 may reduce susceptibility to limitations inherent to active technologies, including ultrasound, CT, fMRI, and other techniques. Future studies are warranted to further validate this technology, investigate its performance across diverse patient populations, and optimize its clinical applicability.

## 5. Conclusions

In conclusion, the results of this first-in-human comparative clinical study demonstrate that non-invasive ICP monitoring with the novel, fully passive Archimedes 02 system can characterize ICP pulse wave morphology and reflect physiological parameters associated with intracranial compliance. The high correlation with invasive recordings, together with recent insights into the mechanical coupling between ICP dynamics and eyeball movements, supports the feasibility of this approach for non-invasive assessment of ICP dynamics. Further refinement and validation across diverse clinical conditions will be necessary, but this technology has the potential to provide a safer and more accessible alternative to current invasive ICP monitoring methods.

## Figures and Tables

**Table 1:** Demographic and clinical characteristics of patients included in the study.

Patient Number	Age	Sex	Pathophysiological Condition	Sedation Method	Ventilation Mode	ICP Sensor	ICP Amplitude, mmHg	GCS	GOS
**1**	40	M	Subarachnoid hemorrhage	RASS −4 (propofol, remifentanil)	MLV (CPAP, spontaneous)	PAR	10.2	14	3
**2**	49	M	Subarachnoid hemorrhage	No sedation	Breathing spontaneously	PAR	7.12	11	5
**3**	73	F	Brain tumor removal	No sedation	MLV (CPAP, spontaneous)	EVD	11.01	14	4
**4**	78	F	Intracranial aneurysm	RASS −5 (propofol, remifentanil)	MLV (CPAP, spontaneous)	PAR	6.64	9	2
**5**	61	F	Subarachnoid hemorrhage	RASS −4 (propofol, remifentanil)	MLV (VC-SIMV)	PAR	3.33	4	3
**6**	68	F	Subarachnoid hemorrhage	RASS −3 (propofol)	Breathing spontaneously	EVD	9.88	9	3
**7**	31	M	Traumatic brain injury (fall)	No sedation	MLV (ASV)	PAR	5.45	5	2
**8**	85	M	Traumatic brain injury (RTA)	No sedation	MLV (ASV)	EVD	13.75	6	1
**9**	81	M	Traumatic brain injury (fall)	No sedation	MLV (ASV)	PAR	0.58	7	2
**10**	64	F	Traumatic brain injury (fall)	No sedation	MLV (ASV)	PAR	4.27	7	2
**11**	51	F	Traumatic brain injury (fall)	RASS −5 (propofol, remifentanil)	MLV(VC-CMV)	PAR	8.4	8	1
**12**	68	M	Brain tumor removal	No sedation	Breathing spontaneously	EVD	7.94	4	1
**13**	52	F	Brain tumor removal	RASS −1 (dexmedetomidine)	Breathing spontaneously	PAR	5.03	15	5
**14**	35	F	Intracerebral hemorrhage	RASS −4 (propofol, fentanyl)	MLV (VC-AC)	EVD	3.34	4	2
**15**	20	F	Intracerebral hemorrhage	RASS −4 (propofol, fentanyl, midazolam)	MLV (VC-CMV)	PAR	4.05	8	5

Abbreviations: M, male; F, female; RTA, road traffic accident; RASS, Richmond Agitation–Sedation Scale—a 10-point scale ranging from +4 (combative) to −5 (unarousable); MLV, mechanical lung ventilation; CPAP, continuous positive airway pressure; VC-SIMV, volume-controlled synchronized intermittent mandatory ventilation; ASV, adaptive support ventilation; VC-CMV, volume-controlled continuous mandatory ventilation; VC-AC, volume-controlled assist control; ICP, intracranial pressure; EVD, external ventricular drainage; PAR, parenchymal intracranial pressure sensor; GCS, Glasgow Coma Scale; GOS, Glasgow Outcome Scale.

**Table 2:** Three-way comparison of averaged invasive ICP, non-invasive ICP (Archimedes 02), and ABP waveforms, showing Pearson’s correlation coefficients, Fisher’s z-transformed values, and P2/P1 ratios calculated from the averaged invasive and non-invasive ICP waveforms.

Patient Number	R(ICP, nICP)	z(ICP, nICP)	R(nICP, ABP)	z(nICP, ABP)	R(ICP, ABP)	z(ICP, ABP)	P2/P1 (ICP)	P2/P1 (nICP)
**1**	0.987	2.515	0.834	1.201	0.794	1.082	0.879	1.046
**2**	0.960	1.946	0.458	0.495	0.609	0.707	1.066	1.099
**3**	0.844	1.235	-	-	-	-	0.516	0.833
**4**	0.854	1.271	0.912	1.539	0.848	1.249	1.287	0.588
**5**	0.981	2.323	0.750	0.973	0.666	0.804	1.483	1.519
**6**	0.766	1.011	0.479	0.522	0.882	1.385	1.013	2.662
**7**	0.932	1.673	-	-	-	-	1.110	1.361
**8**	0.986	2.477	0.562	0.636	0.665	0.802	0.765	0.834
**9**	0.979	2.273	0.817	1.148	0.809	1.124	0.919	1.176
**10**	0.977	2.227	0.876	1.358	0.834	1.201	0.844	1.143
**11**	0.982	2.351	0.465	0.504	0.458	0.495	2.039	1.716
**12**	0.950	1.832	0.501	0.551	0.521	0.578	0.933	1.010
**13**	0.993	2.826	0.504	0.555	0.595	0.685	1.516	1.736
**14**	0.960	1.946	0.819	1.154	0.935	1.697	0.661	0.821
**15**	0.982	2.351	0.556	0.627	0.662	0.796	1.048	1.086

Abbreviations: ICP, averaged invasive intracranial pressure pulse waveform; nICP, averaged non-invasive intracranial pressure pulse waveform obtained with the Archimedes 02 device; ABP, averaged invasive arterial blood pressure pulse waveform; P2/P1, ratio of peak P2 to peak P1; R, Pearson’s correlation coefficient; z, Fisher’s z-transformed value.

## Data Availability

Due to privacy concerns and ethical considerations, access to the clinical data used in this study is restricted. The data are available upon reasonable request, subject to approval by Regional Vilnius Biomedical Research Ethics Committee (rbtek@mf.vu.lt).
